# Virtual Cohorts and Synthetic Data in Dementia: An Illustration of Their Potential to Advance Research

**DOI:** 10.3389/frai.2021.613956

**Published:** 2021-05-17

**Authors:** Graciela Muniz-Terrera, Ofer Mendelevitch, Rodrigo Barnes, Michael D. Lesh

**Affiliations:** ^1^Edinburgh Dementia Prevention Group, University of Edinburgh, Edinburgh, United Kingdom; ^2^Syntegra, San Carlos, CA, United States; ^3^Aridhia Informatics, Glasgow, United Kingdom; ^4^University of California San Francisco, Mill Valley, CA, United States

**Keywords:** AI/ML, virtual cohort, synthetic data, dementia, cohorts

## Abstract

When attempting to answer questions of interest, scientists often encounter hurdles that may stem from limited access to existing adequate datasets as a consequence of poor data sharing practices, constraining administrative practices. Further, when attempting to integrate data, differences in existing datasets also impose challenges that limit opportunities for data integration. As a result, the pace of scientific advancements is suboptimal. Synthetic data and virtual cohorts generated using innovative computational techniques represent an opportunity to overcome some of these limitations and consequently, to advance scientific developments. In this paper, we demonstrate the use of virtual cohorts techniques to generate a synthetic dataset that mirrors a deeply phenotyped sample of preclinical dementia research participants.

## Introduction

In 2019, the World Health Organization reported that around 50 million people had dementia and there are nearly 10 million new cases every year (Alzheimer's disease facts and figures, 2020). Dementia, a disease that affects an ever increasing proportion of the world's population, has ubiquitous deleterious effects on individuals which range from important deterioration of individuals and family members' well-being, to huge economic costs and considerable demand of public health services (Prince et al., 2014) making the case for advancing our understanding of dementia a critical priority for society. Scientific progress often results from synergetic interactions and joint efforts of academia, industry and lay members of society. Yet, in dementia, these efforts and interactions have not yet succeeded in the development of an effective treatment or cure for the disease.

Observational studies and clinical trials are important resources in research. Observational studies are mostly used to better understand the epidemiology of the disease and randomized clinical trials are the gold standard for testing efficacy of new drugs. In the context of dementia research, features of these studies have been discussed in the literature, and researchers have highlighted some challenges and methodological limitations of both types of studies (Ritchie et al., 2015). For instance, amongst other important features, limitations of the studies include their deficient design, the erroneous selection of individuals into the studies and focusing on erroneous outcomes. In fact, it has been argued that some of these features may partially explain the lack of success in the development of disease-modifying treatments for dementia (Anderson et al., 2017).

Importantly, both types of studies heavily depend on the participation of individuals who generously donate their time, data and specimens for research. In recent years, positive developments in research practices have resulted in increased engagement of research participants in most aspects and stages of research studies. However, the burden on participants can become substantial, and key barriers still exist that discourage some individuals from becoming involved in dementia research (Cardona-Morrell et al., 2017). For instance, concerns about data confidentiality and individuals' privacy are important worries that prevent some individuals from engaging in research (Grill and Karlawish, 2010). Furthermore, in some contexts such as low and middle income countries, where the number of dementia cases are expected to increase at a rate faster than in high income countries research and recruitment of study participants is even more challenging for various factors, including the lack of economic resources and cultural barriers (Ferri and Jacob, 2017). Consequently, the recruitment of individuals into dementia studies can be a challenging task for researchers, and as a result, studies may not have the optimal design to answer questions of interest.

Various practices further limit access and use of existing data sources by researchers interested in conducting research, although recent initiatives are making efforts to change practice. Limitations include, amongst others, limited data documentation, possessiveness of data and even limitations for data transferring and storage (Deetjen et al., 2015).

Virtual cohorts are digital non-identical, yet highly similar, synthetic data records that preserve the statistical properties of the original data, that pose new opportunities to advance dementia research. For instance, they may be used for the simulation of clinical trials with specific designs (Koval et al., 2019), to augment the size of datasets to improve the power to detect effects of interest, or of under-represented groups in a study (for example, ethnic minorities). They may also be used when access to person-level data is challenging, offering additional opportunities to analyse data that preserves the data features whilst not using the real data. Indeed, virtual cohorts have been used in other research areas such as heart models (Niederer et al., 2020), and molecular and clinical analysis of cancer virtual cohorts (Guan et al., 2020).

Yet, virtual cohorts are not incorporated in mainstream dementia research. The aim of this paper is to demonstrate the use of a virtual cohort to answer a commonly posed question in dementia research about factors associated with amyloid positivity using data from the European Prevention of Alzheimer's Dementia Longitudinal Cohort Study [EPAD LCS, Ritchie et al. (2016)].

## Materials and Methods

### EPAD LCS

The European Prevention of Alzheimer's Dementia (EPAD) project was initiated in January 2015 and is funded by the Innovative Medicines Initiative (Ritchie et al., 2020). The EPAD LCS had as its primary objective to be a readiness cohort for the EPAD Proof of Concept Trial and its secondary objective was to use the data generated for disease modeling. Based on this secondary objective, we selected this dataset to illustrate our methodology.

Research participants were eligible for inclusion if they were over the age of 50 and should not have a diagnosis of dementia. They must have also been deemed suitable in principle for later inclusion in a clinical trial and therefore should not had any medical or psychiatric disorders which would normally exclude people from such trials.

At baseline and subsequent assessments, research participants completed the entire protocol of assessments, which included the assessments of various domains including basic demography, fluid biomarkers, genetics, lifestyle, clinical, and psychiatric assessment, neuropsychiatric symptoms, function, cognition, and neuroimaging. For full details about the study protocol, please see Solomon et al. (2019).

Specifically, sociodemographic information included data on participants' age, sex, and education (years). The only biomarkers data collected in the EPAD LCS study are CSF (cerebrospinal fluid) ABeta_1_42, Tau and Phosphorylated Tau (p-tau and t-tau). Using data from CSF ABeta_1_42, a threshold of 1,000 pg/ml was agreed upon to define amyloid positivity.

With regards to cognition, participants completed the Repeated Battery for the Assessment of Neuropsychological Status [RBANS, Sullivan et al. (2018)] and they were all tested for ApoE status. Sampling preparation and storage details can be found in the EPAD lab manual (available online at http://www.ep-ad.org). Similarly, participants were asked about their family history of dementia, and their height and weight were also measured.

### Analytical Methods

From the original EPAD analytical database, the EPAD cohort of 1,500 patient records (EPAD V1500) were extracted, including age, sex, ethnicity, years of education, height, weight, family history of dementia, as well as the p-tau, t-tau, ABeta_1_42, and RBANS. We performed pre-processing as follows:

- ABeta_1_42 in some cases has strings such as “>1,700” or “ <200”; those have been transformed to 1,701 and 199, respectively
- We created a new binary variable “abeta < 1,000,” with a value of 0 if the ABeta_1_42 has a value <1,000 and 1 otherwise
- We computed body mass index (BMI) from height and weight using the standard formula BMI = weight (kg)/height^2^ (cm)
- We computed a binary indicator of family history of dementia that took the value of 1 if either father or mother had had a history of dementia and they were the biological father or mother (respectively).

Using this cohort, we used Syntegra's synthetic data engine to create a synthetic dataset which has the same schema and validated the statistical fidelity of the synthetic data with the real data.

For validation we applied a comprehensive set of methods as described next.

#### Univariate Marginal Distributions

We compared univariate (marginal) distributions of the variables between real and synthetic, and used the KS-statistics^1^ as a goodness-of-fit metric for numeric variables and the KL-divergence^2^ as a goodness-of-fit metric for categorical variables. These two statistics are of statistical fidelity between the real and synthetic variables. When the real distribution matches the synthetic distribution, we expect

- The KS-statistics *p*-value to be high (>0.05), and the closer to 1.0 the better; this is a bit non-intuitive at first and is because in this case our null hypothesis is that the two distributions are the same, so a low *p*-value represents a high likelihood of the non-null hypothesis
- The KL-divergence to be low (as close to zero as possible). Intuitively, KL-divergence reflects the information loss when the distribution of the variable in synthetic data is used to approximate the distribution in the real data, which doesn't lend itself to an easy interpretation in terms of a clear threshold of “strong fidelity” vs. “weak fidelity” in the same way KS-Statistics does; nevertheless, one can compare the KL-divergence of a given variable over multiple synthetic datasets and lower values mean better statistical fidelity.

#### Pairwise Variable Correlation

We measured pairwise correlation between each pair of variables in our dataset, using the Pearson correlation coefficient, and compared the resulting heatmap of correlations between real and synthetic. Furthermore, we computed the L1-norm of the difference in correlation between real and synthetic cohorts, in a metric defined as: PCD-L1 = ||*Corr*(*real*) − *Corr*(*syn*)||_1_. Naturally, a low value of PCD-L1 indicates small average difference in the correlation values between real and synthetic, and thus high statistical fidelity of the synthetic data with the real.

#### Predictive Model Performance

To measure how well predictive models perform if trained on the virtual cohort, we trained a predictive model for amyloid-positivity (ABeta_1_42 < 1,000) using some of the other variables (excluding other outcome variables) as predictors; we trained two models - one using the real data to train the predictive model, and another using the virtual cohort.

In both cases we used:

1. Gradient Boosted Trees (using the LightGBM^3^ python library), a commonly used modeling technique.
2. Logistic regression.

We measured the predictive performance of both models using the area under the ROC-curve (AUC-ROC) and compared the models. For high statistical fidelity between the real and virtual cohort, we expect the AUC of the predictive model built with the synthetic data to be similar to that built with the real.

For the GBM models, we further look at the feature importance metric, using SHAP (SHapley Additive exPlanations) values^4^, for our gradient boosted trees model, where we expect to see relative stability in feature ranking (and understanding that this can change due to the inherent randomness of the GBM algorithm). We use normalized Discounted Cumulative Gain^5^ (nDCG) to measure the difference in ranking.

#### Discriminator AUC

Inspired by generative adversarial networks, another effective metric for multivariate fidelity is the “discriminator AUC.” Concretely, we built a classification model trained to discriminate between patient records in the real data vs. the synthetic data; using ROC-AUC as a measure of performance for this discriminator model, a virtual cohort with high statistical fidelity to the real cohort will result in a ROC-AUC value for this discriminator model that is close to 0.5 (representing random decision classifier), whereas low fidelity is reflected with ROC-AUC values closer to 1.

#### UMAP-Based Dimensionality Reduction

UMAP^6^ is a modern dimensionality reduction technique that is often used to understand clustering of points in high dimensional space. It is informative to inspect the 2D projection of real and synthetic data after transforming the high dimensional space into two dimensions via UMAP, and visualize the various clusters formed; specifically, we expect high fidelity synthetic data to demonstrate broad coverage, such that in every cluster for the real data, there is enough number of synthetic data points.

#### Distance-To-Closest-Row (DCR)

We demonstrate privacy of the synthetic data by measuring the distance of each synthetic record to its closest record in the real dataset. Concretely we visualize two distributions: (1) the distance between each synthetic record and its closest real record and (2) the distance between each real record and its closest real record (excluding itself of course). For this purpose, we implement a distance function between two records. Given two records X = (X_0_, X_1_, …, X_p_) and Y = (Y_0_, Y_1_, …, Y_p_), we bin event numeric variables into 25 bins (thus, numeric values turn into a categorical value), and compute the distance between X and Y is computed as:

$$d\left(X,Y\right)=\sum_{i=1}^{p}d\left(X_{i},Y_{i}\right)=\sum_{i=1}^{p}\{\begin{array}{c} 0\;if\;X_{i}=Y_{i}\\ 1\;otherwise \end{array}$$

When DCR values are close to zero, that means synthetic records are similar to the original records resulting in reduced privacy protection. Importantly, the original data may already include significant similarity between records or even explicit duplicates, and so we visualize DCR of real-vs.-real and real-vs.-synthetic to fully understand any reduction in risk.

## Results

Descriptive measures of the set of key variables used in the analyses from the EPAD V1500 sample and the synthetic dataset are shown in Table 1.

**Table 1:** Descriptive statistics of the EPAD V1500 sample used and the synthetic data generated.

**Variable**		**Real data**		**Synthetic data**		
		**Missing**	**Overall**	**Missing**	**Overall**	***p*-value**
						**(difference in means**
						**or proportions)**
*N*			1,498		1,498	
			**Mean (SD)**		**Mean (SD)**	
Height		41	166.7 (9.3)	38	167.0 (8.5)	0.32
Weight		37	73.4 (14.5)	38	74.5 (14.0)	0.42
BMI		41	26.3 (4.5)	38	26.6 (4.5)	0.07
P-tau		236	19.0 (10.2)	320	19.6 (10.1)	0.11
T-tau		236	219.6 (93.1)	320	227.1 (95.5)	0.06
ABeta_1_42		235	1247.4 (420.8)	320	1279 (421.1)	0.94
Abeta_calc		1,130	2276.5 (633.0)	1130	2283.4 (634.7)	0.88
Rad_pct		106	5681.2 (1055.3)	51	5800.7 (1131.7)	0.11
Age		0	65.6 (7.2)	0	65.5 (7.4)	0.78
Edu_years		8	14.5 (3.7)	1	14.3 (3.8)	0.14
Rbans_total		62	103.4 (13.7)	38	104.5 (13.0)	0.21
			***n*** **(%)**		***n*** **(%)**	
Sex	Female	0	852 (56.9%)	0	969 (64.7%)	0.06
	Male		646 (43.1%)		529 (35.3%)	0.06
Ethnicity	Asian	359	2 (0.2%)	322	1 (0.1%)	0.56
	Black		2 (0.2%)		2 (0.2%)	0.99
	Caucasian/White		1,128 (99%)		1,165 (99.1%)	0.43
	Other		1 (0.1%)		2 (0.2%)	0.90
	Hispanic		1 (0.1%)		3 (0.3%)	0.31
	Latin American		1 (0.1%)		2 (0.2%)	0.56
	Mauricienne		1 (0.1%)		0 (0%)	0.31
	Moroccan		1 (0.1%)		1 (0.1%)	0.90
	South East Asian		1 (0.1%)		0 (0%)	0.31
Family history	No	0	575 (38.4%)	0	635 (42.4%)	0.08
	Yes		923 (61.6%)		863 (57.6%)	0.15
ABeta_1_42 < 1,000	No	235	855 (67.7%)	320	830 (70.5%)	0.56
	Yes		408 (32.3%)		348 (29.5%)	0.14
ApoE	e2/e2	178	3 (0.2%)	123	5 (0.4%)	0.34
	e2/e3		110 (8.3%)		106 (7.7%)	0.56
	e2/e4		43 (3.3%)		58 (4.2%)	0.21
	e3/e3		709 (53.7%)		620 (45.1%)	0.05
	e3/e4		404 (30.6%)		526 (38.3%)	0.05
	e4/e4		51 (3.9%)		60 (4.4%)	0.51

We evaluated the synthetic data generated applying the Syntegra methodology to the EPAD V1500 dataset according to the instruments described before. Unless stated otherwise, in all the following experiments, the number of synthetic samples generated is identical to the number of samples in the real dataset, although synthetic data generation allows for generation of any desired number of synthetic records. In all visualizations (if appropriate) we use the color blue to represent real data and the color green to represent synthetic data.

### Univariate Marginal Distributions

**Figure 1:**
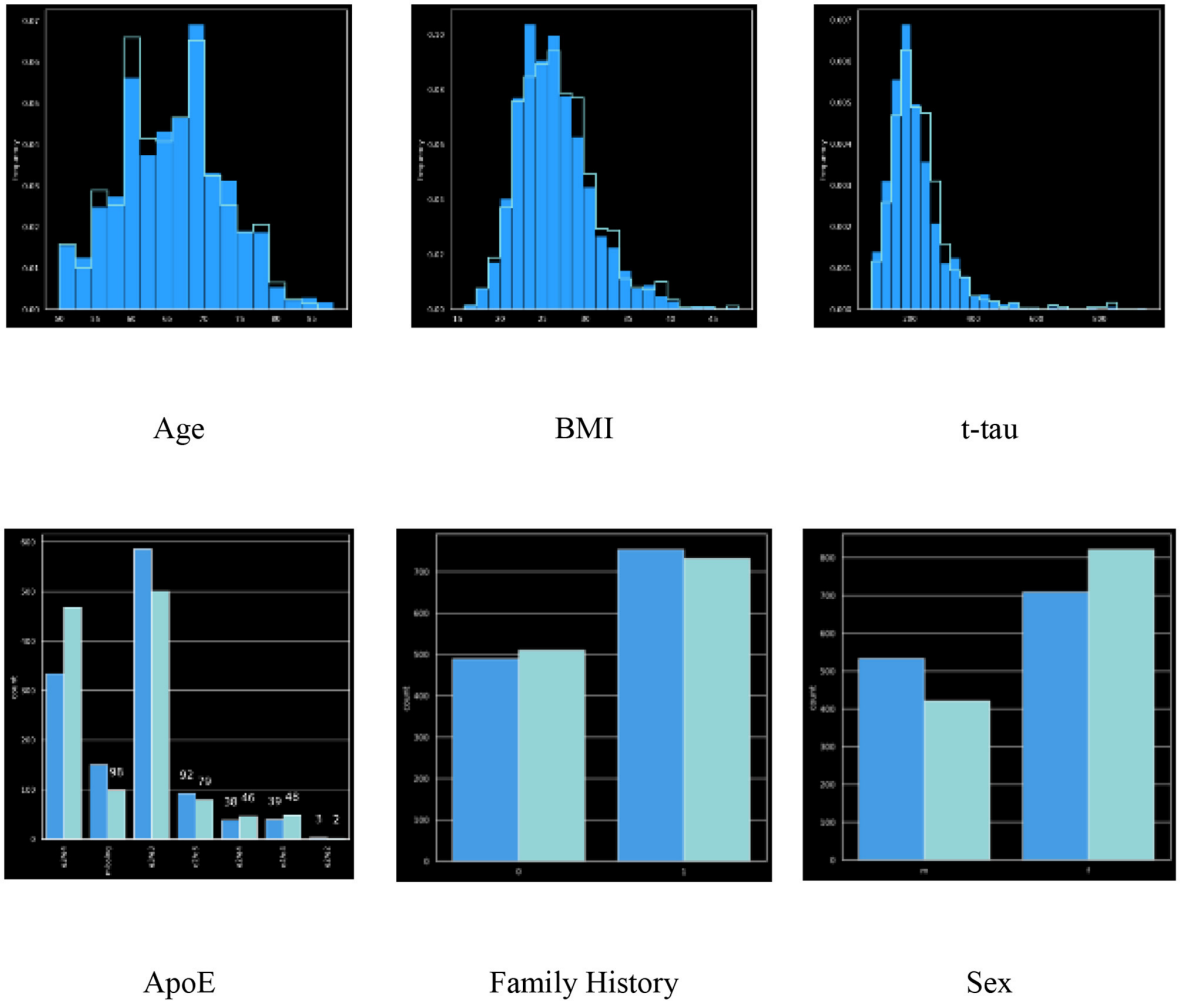
Univariate distributions of Age, BMI, t-tau, ApoE gene, Sex, and family history.

We generated univariate distributions for selected variables in the dataset. Figure 1 displays the univariate distributions for these selected variables in the dataset; a summary of the KS-statistic (for categorical variables) and KL-divergence (for numeric variables) are shown in Tables 1, 2.

**Table 2:** Measures of statistical fidelity of the synthetic data with the real data.

**Numeric variable**	**KS-statistic *p*-value**	**Categorical variable**	**KL-divergence**
ABeta_1_42	0.4175	Abeta_1_42 < 1,000	0.0344
Abeta_calc	0.9999	Apoe4_result	0.0345
Age	0.9999	Ethnicity	0.0031
Body mass index	0.9999	Family history of dementia	0.0005
Education (years)	0.9999	Sex	0.0175
Height (cm)	0.9999		
Weight (kg)	0.9999		
p-tau_result	0.9991		
t-tau_result	0.9980		
rad_pct	0.9999		
Rbans score _total	0.9870		

### Pairwise Variable Correlation

**Figure 2:**
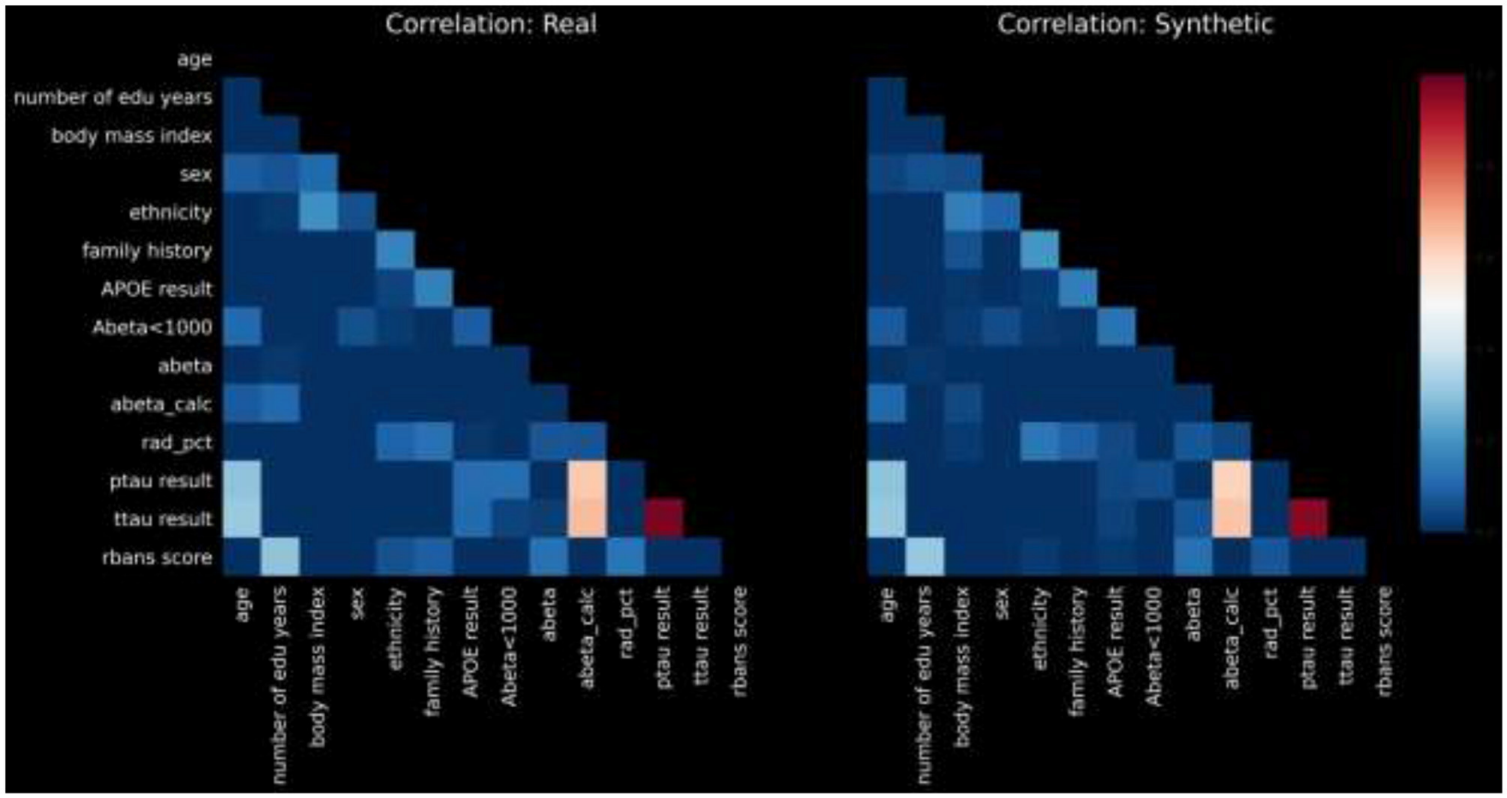
Pairwise distributions.

For the sake of brevity, we present pairwise correlations in graphical form. Figure 2 displays a heatmap of pairwise correlations for real data compared to the synthetic data.

The calculated PCD-L1 is 0.0367, a value that indicates small average difference in the correlation values between real and synthetic, and thus high statistical fidelity.

### Predictive Model Performance

**Figure 3:**
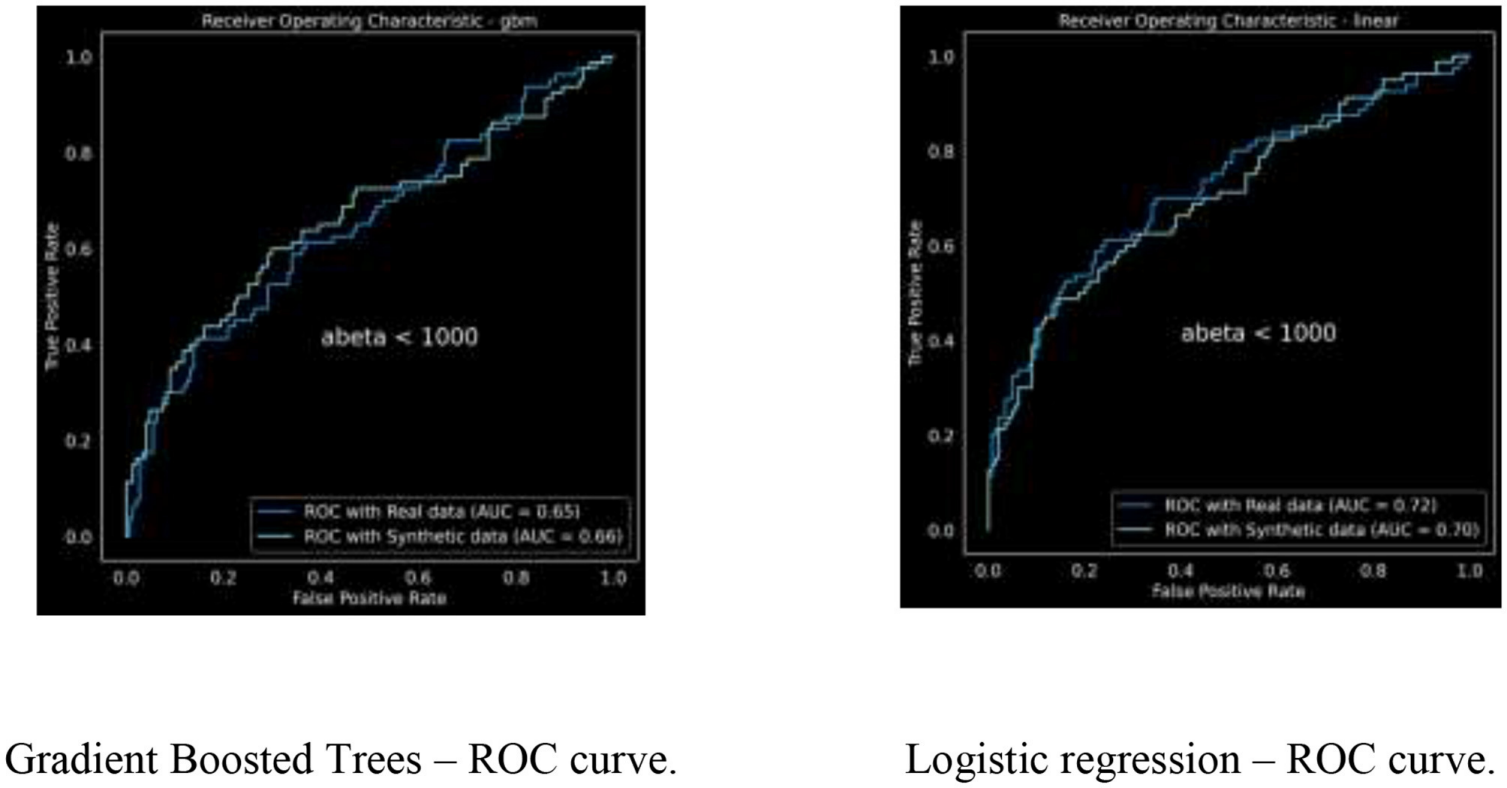
Receiver operating curves for real vs. synthetic (single run).

We trained a gradient boosted trees classifier to predict ABeta_1_42 < 1,000, which is an indicator for dementia. We tuned the model parameters to achieve the best model performance using 50 iterations of hyper parameter tuning. The resulting Receiver Operating Curve (ROC) and area under the ROC (or C-statistic) are in Figure 3.

The ROC curves are demonstrating similar characteristics; we ran this analysis multiple times with varying random initialization each time, resulting in

- For GBM: AUC for real data is 0.6385 ± 0.0109 and for synthetic is 0.6745 ± 0.0096
- For Logistic regression: AUC for real data is 0.7232 ± 0.0034 and for synthetic is 0.7032 ± 0.0028.

### Feature Importance Ranking

**Figure 4:**
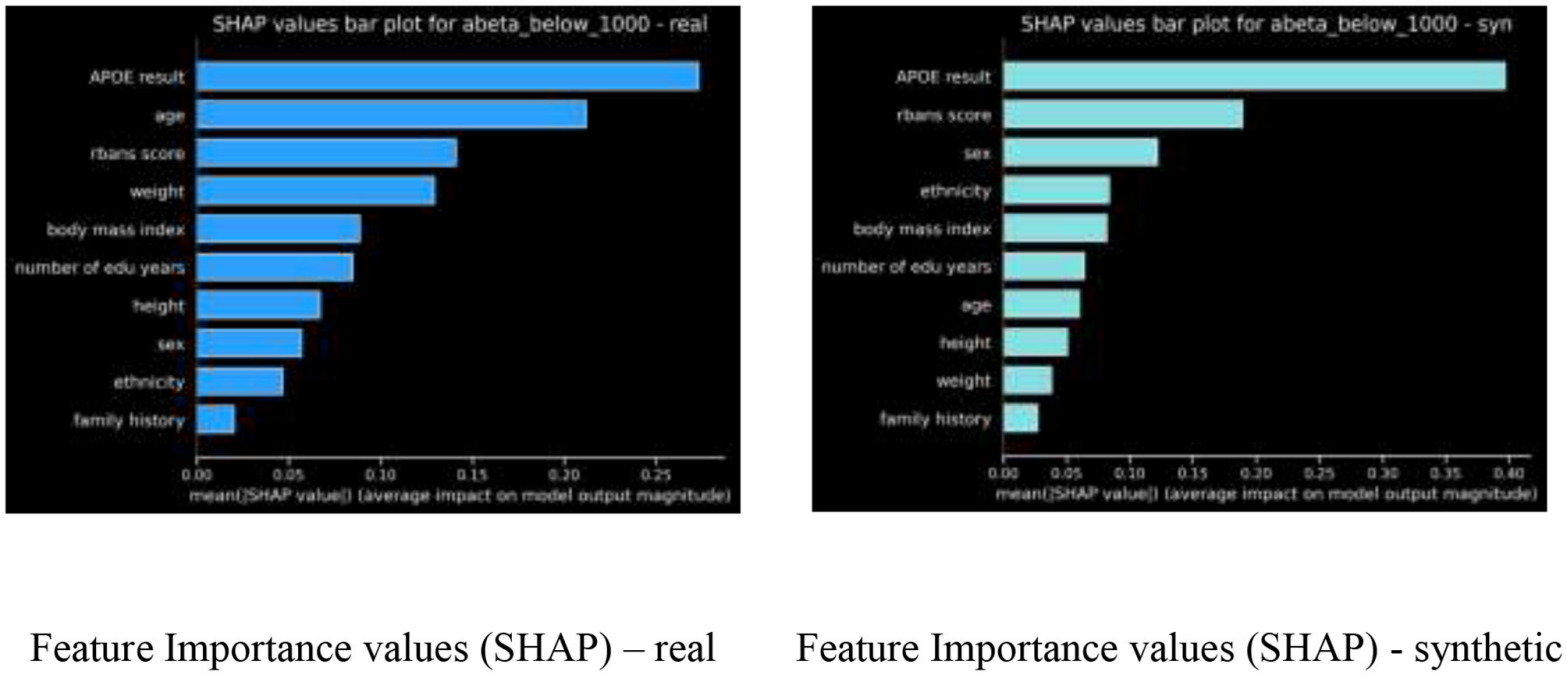
Feature importance using SHAP values compared.

For the Gradient boosted trees model, we compared the feature importance ranking, using SHAP values, of our real model vs. the feature ranking of the synthetic model, as shown in Figure 4.

The nDCG metric comparing these two feature rankings is 0.9244.

### Discriminator AUC

The calculated discriminator AUC, as described in the “Methods” section above resulted in 0.5320 ± 0.0339 which is close to the desired level of 0.5, reflecting good statistical match between real and synthetic.

### UMAP Dimensionality Reduction

**Figure 5:**
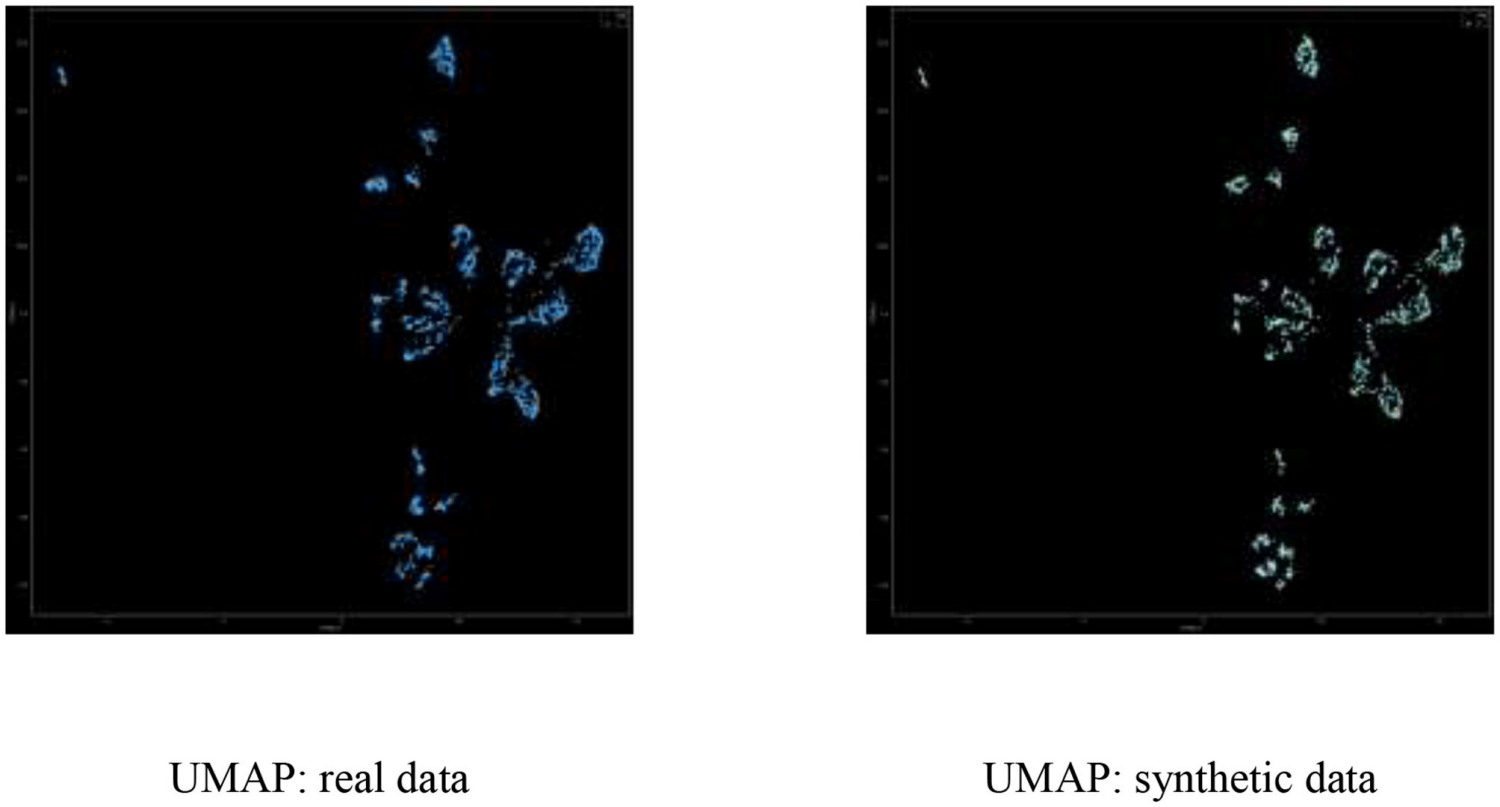
UMAP visualization of real (left) vs. synthetic (right) data points in 2D.

Figure 5 displays the UMAP real-vs.-synthetic 2D scatterplot, demonstrating good coverage of real vs. synthetic in all clusters.

In calculating the UMAP visualization, we used the open source UMAP python package umap-learn^7^, with n_neighbors = 50 and min_dist = 0.1. Importantly, umap is trained on the real data and both real and synthetic data is transformed to the 2D dimension using the same learned model.

### Distance-To-Closest-Row (DCR)

**Figure 6:**
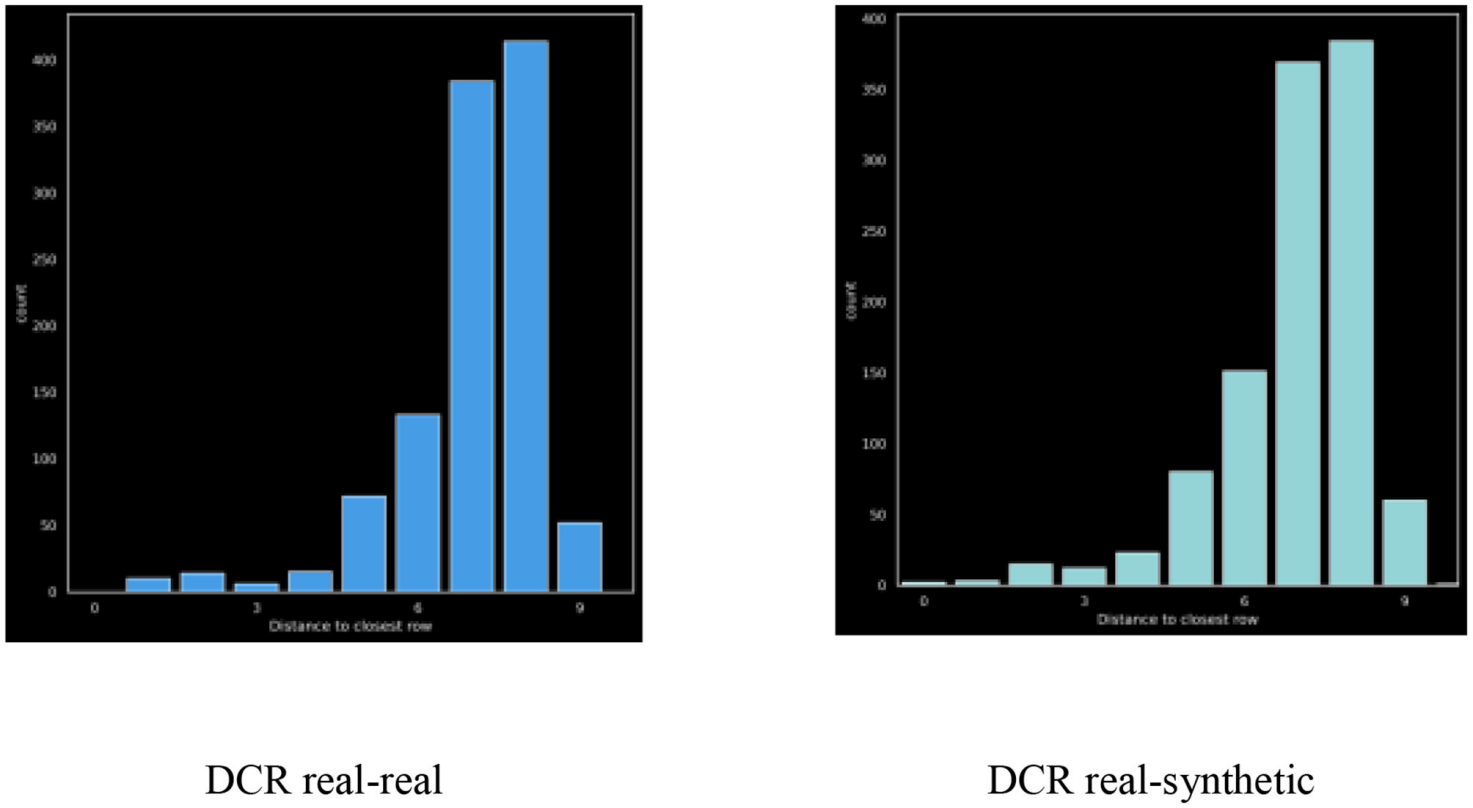
DCR – real on the left (blue), synthetic on the right (green).

Figure 6 also depicts the DCR distribution of the real vs. synthetic data.

The ratio between mean-dcr values for the real and synthetic data provides a quantitative measure of privacy. For this dataset the ratio was: 0.9995 which is excellent, although we do see a slight shift to the left in the synthetic distribution as compared to the real.

## Discussion

We demonstrated the use of synthetic data generated using the Syntegra methodology to produce a virtual cohort of non-identical digital records that preserve the statistical properties of the EPAD V1500 dataset. We showed the fidelity of synthetic data generated, reporting a series of commonly used indices that consistently revealed high degree of similarity at the individual level data between the original dataset and the virtual cohort at the individual level data.

Our work, albeit being an application of modern computational approaches without aiming to answer any specific clinically relevant question, is a clear illustration of the advantages that virtual cohorts via synthetic data generation offer to the research community. Virtual cohorts represent quick and cost-effective opportunities to advance research, overcoming several of the existing hurdles that slow down or even impede scientific developments. For instance, by using virtual cohorts researchers would avoid lengthy and time-consuming data transfers, which would become unnecessary and obsolete, whilst having access to data that preserves the properties of the original datasets of interest whilst also preserving confidentiality and data privacy.

Notably, the generation of virtual cohorts by demand, that is, the generation of tailored synthetic datasets generated for answering specific questions, is another advantage of this technique. However, its feasibility needs to be further explored and may require appropriate documentation registering all steps taken for later external data quality examinations.

The aim of our work was to illustrate, via a specific example, how an advanced synthetic data generation technique could produce a dataset that preserves all features of the true cohort. We show the similarities between the real and the synthetic cohort in a cross-sectional setting. Although we do not demonstrate the use of virtual data in longitudinal designs, techniques exist that permit similar work in these more complex designs, and the recent release by Aridhia and EPAD of the EPAD V.IMI, that contains longitudinal data for over 2,000 participants, can be used for this purpose.

In the very near future, researchers developing and using virtual cohorts in research areas such as dementia research will need to engage with regulatory institutions and industry to explore their systematic use and acceptance of results generated using virtual cohorts. Most importantly, the community still needs to engage with participants to better understand their view regarding the use of their data for the implementation of this approach. Clearly, efforts to continue collecting data need to continue and be further supported as new datasets will always be necessary to answer questions that emerge as scientific knowledge advances. We are not advocating for stopping data collections of new data. Instead, we advocate for the exploration of new opportunities that virtual cohorts offer to maximize the utilization of existing datasets whilst overcoming some of the hurdles that currently disrupts science progression.

## Data Availability Statement

The datasets presented in this study can be found in online repositories. The names of the repository/repositories and accession number(s) can be found below:

Synthetic data: https://doi.org/10.34688/fpv7-b321

EPAD data: http://ep-ad.org/erap/.

## Ethics Statement

The studies involving human participants were reviewed and approved by NHS Health Research Authority. For details see: https://www.hra.nhs.uk/planning-and-improving-research/application-summaries/research-summaries/epad-longitudinal-cohort-study/. The patients/participants provided their written informed consent to participate in this study.

## Author Contributions

GM-T drafted the paper and contributed to the discussions on results. OM and ML performed the analysis and contributed to the manuscript. RB contributed to the manuscript and oversaw the project. All authors contributed to the article and approved the submitted version.

## Conflict of Interest

OM and ML are employed by the company Syntegra. The remaining authors declare that the research was conducted in the absence of any commercial or financial relationships that could be construed as a potential conflict of interest.

## Footnotes

^1^Note that the Chi-Squared metric is also a good metric in this case.

^2^https://en.wikipedia.org/wiki/Kullback%E2%80%93Leibler_divergence

^3^https://github.com/microsoft/LightGBM

^4^https://github.com/slundberg/shap

^5^https://en.wikipedia.org/wiki/Discounted_cumulative_gain

^6^https://arxiv.org/abs/1802.03426

^7^https://pypi.org/project/umap-learn/
